# Metastasis Inhibition

**DOI:** 10.3390/ijms24087123

**Published:** 2023-04-12

**Authors:** Masa-Aki Shibata, Kohei Taniguchi

**Affiliations:** 1 Department of Anatomy and Cell Biology, Division of Life Sciences, Osaka Medical and Pharmaceutical University, 2-7 Daigaku-machi, Takatsuki 569-8686, Osaka, Japan; 2 Translational Research Program, Department of General and Gastroenterological Surgery, Osaka Medical and Pharmaceutical University, 2-7 Daigaku-machi, Takatsuki 569-8686, Osaka, Japan; kohei.taniguchi@ompu.ac.jp

Cancer metastasis is a common biological phenomenon observed in malignant tumors that can lead to death in affected individuals. Therefore, effective antimetastatic therapy and chemopreventive treatments are necessary to minimize the risks of metastasis. The pathobiology of metastasis greatly differs between cancer types. Various microenvironmental factors, genetic events, and cytokines/chemokines contribute to cancer metastasis. Therefore, to achieve successful inhibition of metastasis, these complex mechanisms must be blocked.

The process of cancer metastasis can be classified into different stages beginning from local invasion, intravasation, and circulation in the bloodstream or lymphatic flow to extravasation, colonization, and metastasis. In other words, the dissemination of cancer cells occurs in various ways: local and direct invasion and/or migration via blood and lymphatic vessels. The few cancer cells that complete all these steps can colonize distant sites, i.e., accomplishment of metastasis. Therefore, therapeutics targeting responsible factors at each stage can be considered as potential therapies against metastasis.

This Special Issue, “Metastasis Inhibition,” organized by the International Journal of Molecular Sciences, comprises eight contributions: five original articles and three reviews, all of which provide new information about cancer metastasis inhibition. Clinical studies in breast cancer cohorts showed high expression of miR-143 and prolonged survival [1], while miR-34a suppressed cell proliferation and induced apoptosis. However, miR-143 did not show a favorable improvement in survival due to the promotion of epithelial–mesenchymal transition (EMT), highlighting the complexity of cancer biology [2]. In addition, the tumor G2M score has been reported as a potential predictive tool for cyclin-dependent kinase inhibition therapy [3]. The NKX6.1 (NK homeobox 1) may be a potential therapeutic molecule for colorectal cancer [4]. These molecules and methods hold promise for the future. The article in this Special Issue also showed the possibility of using AI (artificial intelligence) systems to identify effective personalized antitumor drugs for individual patients by using circulating tumor cells (CTCs) in the blood [5]. Three reviews have been included in this Special Issue, and substantial information can be acquired from these regarding strategies for the control of metastasis. In one of the reviews, extracellular vesicles (EVs) are discussed as a diagnostic tool for a new era and a potential new factor for metastasis inhibition [6]. Since EVs have unlimited possibilities, it impossible to negate their potential outright. Other review articles discuss metastasis of breast cancer to the bone [7] and lung cancer to the brain [8], along with relevant therapeutic approaches. A summary of each article is provided below.

NKX6.1 acts as an oncogene against lung cancer [9]. However, it has also been shown to act as a metastasis suppressor against prostate cancer [10]. Chung et al. [4] showed that NKX6.1 represses tumorigenesis, metastasis, and chemoresistance in colorectal cancer. In this study, the authors demonstrated that NKX6.1 suppresses tumorigenic and metastatic potential both in vitro and in vivo. Furthermore, a comprehensive analysis using RNA sequencing technology was performed to demonstrate the mechanism by which NKX6.1 exerts its tumor-suppressive function. They observed differentially expressed genes (DEGs) associated with cell migration, response to a drug, transcription factor activity, and growth factor activity, suggesting that these DEGs are involved in the inhibitory function of NKX6.1 in cancer invasion and metastasis. Their results demonstrated that NKX6.1 functions as a tumor suppressor and enhances chemotherapy sensitivity in colorectal cancer, indicating that NKX6.1 could be a potential molecule for therapeutic strategies.

Tokumaru et al. [1] reported that higher expression of miR-143 is associated with a favorable tumor immune microenvironment and better survival in estrogen receptor (ER)-positive breast cancer. It is well known that miR-143 exerts its antitumor function by targeting the KRAS signaling pathway in gastric [11], colorectal [12], and renal cell carcinomas [13]. The authors used two published breast cancer cohorts from The Cancer Genome Atlas (TCGA) and Molecular Taxonomy of Breast Cancer International Consortium (METABRIC) to investigate correlations among miR-143 expression, ER, immune cells in the microenvironment, and survival rates. They found that high expression of miR-143 in cancer cells in patients with ER-positive breast cancer may generate a favorable tumor immune microenvironment, leading to improved survival.

Tokumaru et al. [2] also analyzed the relationship between miR-34a expression and survival rates in breast cancer cohorts. miR-34a is a tumor-suppressive microRNA that is known to be involved in p53 expression and related to the induction of apoptosis [14] and suppression of EMT, exerting anticancer effects [15]. Using two breast cancer cohorts, METABRIC and TCGA, the authors analyzed the relationship between miR-34a expression and survival rates. They observed suppression of cell proliferation, activation in the p53 pathway, and enhancement of apoptosis in breast cancer cells that express high levels of miR-34a. However, they also showed enhanced EMT and failed to demonstrate prolonged survival in large breast cancer cohorts.

Yanagisawa et al. [5] addressed the issue of convolutional neural networks and how they can recognize drug resistance of single cancer cells. It is well known that single or isolated tumor cells circulate in the bloodstream of patients with cancer. The CTCs are an effective tool for diagnosing cancer malignancy [16]. In this study, the authors investigated whether the sensitivity to anticancer drugs can be classified by a deep learning model, convolutional neural network, based on the morphology of cells in culture. They showed that the effect of anticancer drugs could be predicted at the single-cell level. Finally, the authors suggested that, in the future, utilizing precision medicine to identify effective anticancer drugs for individual patients may be possible by extracting CTCs from blood and classifying them using an AI system.

Oshi et al. [3] analyzed the relationship between the G2M pathway gene activity and metastasis using gene expression data from a total of 4626 samples across 12 human breast cancer cohorts. Clinically aggressive features and survival in ER-positive/HER2-negative breast cancer were significantly correlated. Higher G2M scores of metastatic tumors were also significantly associated with worse survival. High-scoring primary and metastatic tumors showed increased antitumor immune cell infiltration. Tumor G2M score was also associated with therapeutic response to systemic chemotherapy in ER-positive/HER2-negative breast cancers. The authors suggested that tumor G2M score predicts the response to cyclin-dependent kinase inhibition therapy.

Kogure et al. [6] extensively reviewed EVs in cancer metastasis. It has been reported that EVs are involved in cancer progression and metastasis, translocating bioactive molecules such as proteins and miRNAs between cancer cells and other cells in local and distant microenvironments [17]. Clinically, the focus in terms of EVs is on their potential as diagnostic biomarkers, therapeutic targets, or anticancer drug delivery vehicles. The potential therapeutic effects of EVs in cancer therapy are rapidly emerging and are becoming an important area of research. In this review, the biological properties of EVs and their functions are described in greater detail, with a focus on the therapeutic effects of EVs and their utility in the suppression of cancer progression (metastasis).

Breast cancer gives rise to tumor colonies in the skeletal system in approximately 70% of advanced breast cancers, leading to the formation of bone metastases [18]. This condition leads to severe disability and reduced quality of life. Decades of research into agents that act in the bone microenvironment to inhibit the development of bone metastases have resulted in the clinical introduction of several bone-targeting agents (BTAs). These also control bone lesions and reduce the risk of skeletal complications. However, because of the potential toxicity of the long-term administration of these agents, several investigators have studied the early administration of BTAs to prevent the development of bone metastases. In this review, D’Oronzono et al. [7] describe the mechanisms of bone metastasis development in breast cancer and describe strategies for selecting patients at high risk of bone metastasis who are suitable for early BTA treatment.

Brain metastases have been observed in several patients with non-small cell lung cancer (NSCLC), and cell populations with abnormalities such as epidermal growth factor receptor (EGFR) mutations and anaplastic lymphoma kinase (ALK) rearrangements are particularly susceptible to brain metastases [19]. Therefore, brain metastasis with such mutations is well understood. However, little is known about the molecular mechanisms of brain metastasis in patients with other fusion oncogene drivers of NSCLC. In their review, Tan et al. [8] describe the biology of brain metastasis in fusion-driven lung cancers and discuss the efficacy of novel systemic therapies for brain metastasis.

It would be no exaggeration to note that overcoming cancer is equivalent to preventing metastasis. Cancer research is constantly being undertaken by many researchers, and numerous data are being generated. Based on these results, practical methods that lead to the complete prevention of cancer metastasis will emerge in the future. We believe that this Special Issue will contribute to the work of many researchers and will provide ideas for metastasis inhibition research. 
